# Head Lice Infestation in Schoolchildren, in Poland—Is There a Chance for Change?

**DOI:** 10.3390/jcm11030783

**Published:** 2022-01-31

**Authors:** Katarzyna Bartosik, Marzena Janczaruk, Zbigniew Zając, Aleksandra Sędzikowska, Joanna Kulisz, Aneta Woźniak, Anita Jasztal-Kniażuk, Ewa Kulbaka, Andrzej Tytuła

**Affiliations:** 1 Department of Biology and Parasitology, Faculty of Health Sciences, Medical University of Lublin, Radziwiłłowska 11, 20-080 Lublin, Poland; zbigniew.zajac@umlub.pl (Z.Z.); joanna.kulisz@umlub.pl (J.K.); aneta.wozniak@umlub.pl (A.W.); 2 II Chair and Department of General and Gastrointestinal Surgery and Surgical Oncology of the Alimentary Tract, Faculty of Medicine, Medical University of Lublin, 20-081 Lublin, Poland; marzena.janczaruk@gmail.com; 3 Department of General Biology and Parasitology, Faculty of Medicine, Medical University of Warsaw, 02-004 Warsaw, Poland; aleksandra.sedzikowska@wum.edu.pl; 4 Regional Chamber of Nurses and Midwives in Lublin, 20-072 Lublin, Poland; a.jasztal@oipip.lublin.pl (A.J.-K.); a.tytula@oipip.lublin.pl (A.T.); 5 Department of Pediatric Hematology, Oncology and Stem Cell Transplantation, Faculty of Medicine, Medical University of Lublin, 20-093 Lublin, Poland; ewakulbaka@op.pl

**Keywords:** pediculosis capitis, *Pediculus humanus capitis*, head louse, ectoparasites, parasitic infestation

## Abstract

Pediculosis capitis is a current and neglected health issue worldwide. The lack of screening programs contributes to the marginalization of the problem and delays therapeutic measures. Our study aimed to analyze the occurrence of this parasitosis in primary schools in Poland and to determine factors contributing to the persistence of its foci. The research tools were two questionnaires: one for primary school children and the other for school managers. While children answered questions about the epidemiology of pediculosis capitis and expressed their opinion on the hygienic condition of infested persons, the school directors were asked about the occurrence of head lice in schools, preventive measures, and institutions supporting schools in combating the infestation. The survey covered the period 2014–2018. Pediculosis capitis was reported in 87.5% of the schools. The greatest number of cases was reported in the group of 6–9 year-olds (68%). Among 4970 children, 16.7% had no knowledge of head lice; however, 57.1% wanted to increase their awareness of the problem. Campaigns on lice were conducted mainly as a result of emerging pediculosis capitis cases, and most schools could not rely on institutional support. Screening programs and preventive educational campaigns should be part of pediculosis capitis control in Poland.

## 1. Introduction

Schoolchildren are especially susceptible to contagious diseases, including parasitic infections [1,2,3]. This is mainly related to the immaturity of their immune system and poor adaptation to a prolonged stay in confined conditions [4,5]. Diseases caused by ectoparasites spread easily in children staying in large groups [6,7]. One of the most frequently observed diseases of this type is pediculosis capitis caused by hematophagous insects *Pediculus humanus capitis* [8].

Humans are the only hosts of *P. humanus capitis*. This ectoparasite is most often transmitted between these hosts through direct ‘hair-to-hair’ contact. A possible, but much less frequent, route of transmission is the shared use of headgear, combs, and hair brushes [9,10], as head lice are able to survive up to 30 hours away from the host’s body [11]. Moreover, *P. humanus capitis* insects are sensitive to thermal and humidity conditions. The optimal ranges for their growth are 28–29 °C and 70–90%, respectively [12].

**Figure 1:**
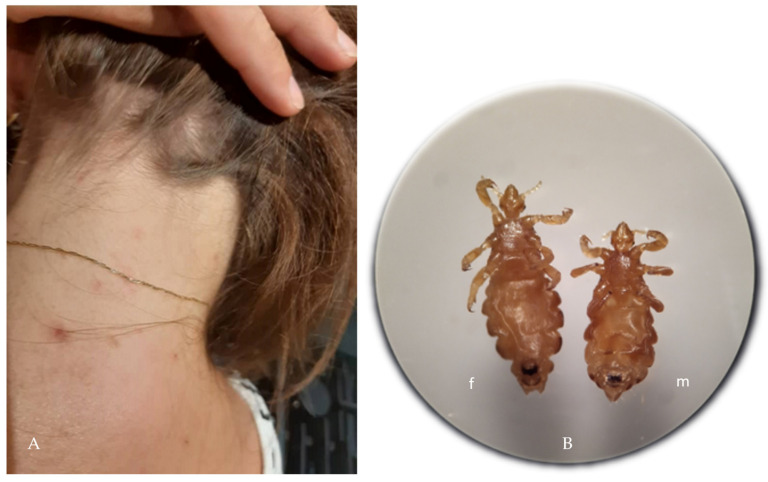
Scalp infestation caused by *Pediculus humanus capitis* accompanied by allergic reaction (**A**); *Pediculus humanus* specimens removed from the patient’s scalp (**B**); f—female; m—male; original magnification 10×, photo by Katarzyna Bartosik.

Lice infestation leads to development of a hypersensitivity reaction to the components of the saliva of these insects appearing after 4–6 weeks in the case of the first infestation or after ca. 2 days in re-infestation [11]. In the area of the hairy skin of the head, and less often on the neck (Figure 1), papular itchy lesions can be observed at the parasite feeding site, which may result in excoriations. Secondary infection with *Staphylococcus aureus* and streptococci (impetiginization) may develop as well. Local enlargement of cervical lymph nodes has been observed in some patients. Severe and chronic infestation may lead to anemia and alopecia [13]. Furthermore, the awareness of lice infection may cause stress and emotional tension, leading to irritability and sleeplessness [14,15].

A particularly important epidemiological issue is the potential but still not fully explored role of *P. humanus capitis* as a vector and reservoir of pathogenic microorganisms. The capability of head lice of transmission of *Rickettsia prowazekii* [16] and proliferation and excretion of *Bartonella quintana* [17] has been experimentally confirmed. Moreover, genetic material of many other pathogens has been detected in this *Pediculus humanus* ecotype. Nevertheless, the role of *P. humanus capitis* as a competent vector of *Borrelia recurrentis*, *Borrelia theileri*, *Yersinia pestis*, *Coxiella burnetii*, *R. aeschlimannii*, *Anaplasma* spp., *Ehrlichia* spp., *Ehrlichia* aff. *mineirensis*, *Acinetobacter* spp., and Moraxellaceae is debatable or yet unknown [18].

Although head lice infestation is an important epidemiological problem in Poland, it is not included in the list of infectious diseases published in the appendix to the Act of 5 December 2008 on prevention and control of infections and contagious diseases in humans. Therefore, it is very difficult to assess the scale of its occurrence in our country. The aim of our study was to conduct extensive research on the prevalence of head lice in schoolchildren in Poland and to determine factors contributing to the occurrence of pediculosis capitis. A rationale for the present investigation was the fact that the previous studies on head lice conducted in our country were less comprehensive [19,20,21,22] or involved other social groups [23,24,25].

## 2. Materials and Methods

**Figure 2:**
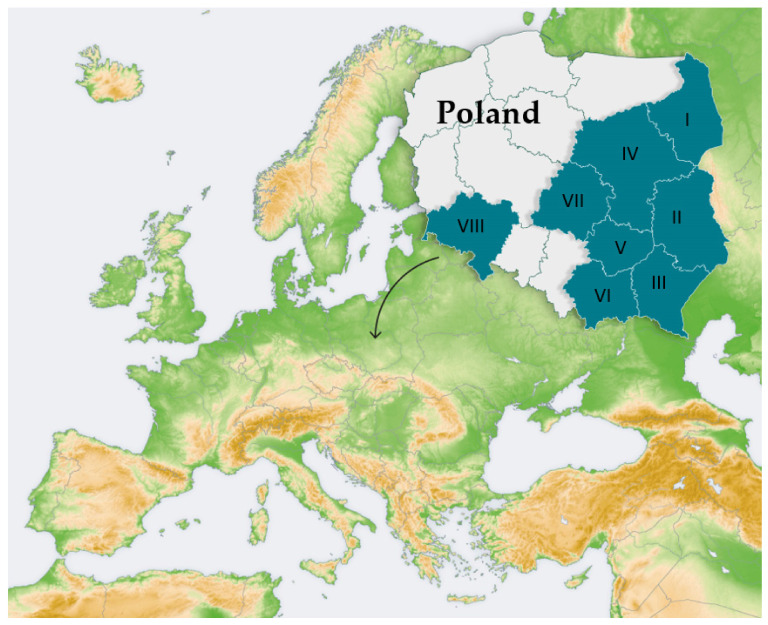
Location of the study area in Europe based on Wikimedia with our modifications (the area where the survey was conducted is marked in dark green). Region I-Podlaskie, *n* = 13, *n* = 194; Region II Lubelskie, *N* = 61, *n* = 2317; Region III Podkarpackie, *N* = 33, *n* = 701; Region IV Mazowieckie, *N* = 19, *n* = 543; Region V Świętokrzyskie, *N* = 6, *n*= 225; Region VI Małopolskie, *N* = 9, *n* = 251; Region VII Łódzkie, *N* = 17, *n* = 413; Region VIII Dolnośląskie, *N* = 10, *n* = 326; *N*- number of schools surveyed; *n*-number of pupils surveyed.

The data subjected to the analysis were obtained in a survey conducted in eight regions in Poland—i.e., Podlaskie, Lubelskie, Podkarpackie, Świętokrzyskie, Łódzkie, Małopolskie, Mazowieckie, and Dolnośląskie Provinces—in 2014–2018 (Figure 2). The research was conducted with the use of two questionnaires: one was addressed to primary school children and the other was filled in by school managers. During the study, basic information about the children (age and sex) was collected. No sensitive data, e.g. on the health status of the respondents and their family members, were collected. The analysis was mainly focused on children’s awareness of pediculosis capitis and their declaration to expand the knowledge. The children were asked to answer several questions about the sources and routes of head lice infection. They were also asked to express their opinion on the hygienic condition of patients and to declare whether and who they would inform about their head lice infestation. The aim of the survey conducted among the managers of the educational institutions was to obtain information on the prevalence of head lice in schools as well as the applied preventive measures and subjects responsible for tackling the problem. They were also asked to specify the sources of support offered to the school employees in the preventive actions and measures for reduction of infestations by the parasite diagnosed in the schoolchildren. The participation in the study was voluntary, and the respondents were guaranteed anonymity.

Pearson’s chi-square test of independence was used to test the relationships between categorical (qualitative) variables. In the case of insufficient numbers of expected values, the chi-square test with Yates’s correction was used for 2 × 2 contingency tables and the ML (maximum likelihood) chi-square test was used for contingency tables larger than 2 × 2. 

Pearson’s chi-square test was used to check the cardinality of responses.

The significance of the differences between two structure indices was tested to compare the responses to the multiple-response questions in two groups, and the Fp test comparing frequencies k was applied in the case of at least three groups.

Due to the lack of normality of data distribution, non-parametric tests were used to check the significance of the differences:

- Mann–Whitney U test—to check the significance of the difference in two groups;

- Kruskal–Wallis test—to check the significance of the difference in at least three groups. The Kruskal–Wallis multiple comparison test was used to distinguish pairs of groups differing significantly.

The test of significance of the differences between two structure indices was used to compare the responses in the two age groups.

A value *p* < 0.05 was considered statistically significant. Statistical calculations were performed using the STATISTICA 10 PL (StatSoft Polska Sp. z o.o., Cracow, Poland) statistical package.

## 3. Results

The survey was conducted in 168 schools, including 50 (29.8%) rural schools, 13 (7.7%) suburban schools, and 105 (62.5%) urban schools. The average number of children in the schools was 392 (from 16 to 1300) (Table 1). 

In terms of the number of schoolchildren, the urban schools were the most numerous and the rural schools had the lowest number of pupils. The Kruskal–Wallis test showed a significant difference in the number of schoolchildren between the types of school (*p* < 0.0001). As shown by the Mann–Whitney U test, there was a significant difference in the number of pupils between pediculosis capitis affected and non-affected schools (*p* = 0.0169). The mean number of pupils was higher in schools reporting head lice than in the non-affected schools (387 ± 285 and 234 ± 183, respectively). The ML chi-square test showed no significant correlation between the school region and the occurrence of head lice (χ^2^ = 10.25; df = 7; *p* = 0.1748).

Cases of pediculosis capitis during the analyzed period (2014–2018) were reported in 147 (87.5%) of the schools. The ML chi-square test showed a significant relationship between the location of the school and the prevalence of head lice (*p* = 0.0196). Cases of head lice infestation were observed most frequently in urban (92.4%) and suburban schools (92.3%) (Table 2).

The mean number of children with head lice per school was 10.2 (from 1 to 87 over 5 years). Cases of pediculosis capitis were noted in children aged from 6 to 12 years, and the greatest number of *P. humanus capitis* infestation was recorded in the group of 6–9 year-olds (68%, *N* = 181), with girls accounting for 54% of the infested children (*N* = 144).

Transmission of *P. humanus capitis* among siblings was recorded in 102 schools (69.4%). In 58.5% of the schools participating in the study, all parents signed consent for inspection of their child’s head. Most schools organized educational campaigns, providing information about head lice. Organization of this type of activities was declared by the management of 160 surveyed institutions (95.2%). Most school authorities declared organizing educational campaigns in response to the diagnosis of pediculosis capitis in the pupils (65.6%, *N* = 105).

The elimination of head lice infestation in the schools was perceived as difficult or rather difficult by the staff of 62 schools (40%) out of the 155 schools that commented on this issue. The ML chi-square test did not show any significant correlation between the region and the difficulty in solving the problem with head lice in the schools (*p* > 0.05).

Educational campaigns were organized most often in schools where the staff declared that head lice control was difficult or quite difficult (37.1%) and least frequently in schools where the staff claimed that pediculosis capitis control was easy or quite easy (Table 3). The majority of the school directors (73.8%, *N* = 124) recognized the need to monitor the occurrence of this parasitosis in the school. The ML chi-square test did not show any significant correlation between the region and the need to monitor head lice in the schools (*p* > 0.05).

Out of 55.9% of schools that declared to receive support in case of pediculosis capitis occurrence, the majority could count on the substantive help of employees of the Sanitary-Epidemiological Station (Table 4).

In total, 4970 children were surveyed in the analyzed schools. Girls constituted the majority of the surveyed group (52.3%, *N* = 2597). Pupils aged 6–12 years accounted for 77.3% (*N* = 3842), and the 13–17-year olds represented 22.7% (*N* = 1094). The age of children in primary schools in Poland is 7–15 years; however, it is possible to admit 6-year-olds to school at the request of parents. Schoolchildren repeating a year also participated in the study, hence the upper limit is shifted. 

The surveyed children are aware of the existence of lice (83.1%, *N* = 4128) and have some knowledge about this parasite. The vast majority knew that they could become infected with head lice from another person (77.5%, *N* = 3851). Slightly more than half of the children indicated that lice can survive—e.g. on a comb, towel, or bedding (57.3%, *N* = 2850). However, when asked about the spread of the parasite, only one third of the respondents (36.9%, *N* = 1836) knew that lice are not transmitted by domestic animals. As indicated by most of the schoolchildren, (58.2%, *N* = 2894) pediculosis capitis can affect any child; however, 26.6% of the respondents (*N* = 1323) indicated that lice infest dirty and neglected children. Only 5% of the respondents would inform their friends about their head lice infestation.

Slightly more than half of the surveyed schoolchildren (57.0%, *N* = 2833) gave a positive answer to the question about their willingness to improve their knowledge of head lice. Pearson’s chi-square test showed a significant relationship between the sex of the children and the declaration of willingness to extend their knowledge of head lice (*p* = 0.0022). In comparison with the boys, the girls represented a higher percentage in the group of schoolchildren declaring their willingness to participate in educational programs focused on pediculosis capitis (59.5% and 55.2%, respectively). Pearson’s chi-square test showed a significant relationship between the age of the children and the declaration of willingness to extend their knowledge of head lice infestation (*p* < 0.0001). In the group of children aged 6–12 years, the percentage of those declaring their willingness to extend their knowledge was higher than among the older schoolchildren (62.9% and 38.6% respectively). The percentage of correct answers was not correlated with the age of the schoolchildren, as they were given by either the younger or older schoolchildren, depending on the question. 

## 4. Discussion

Human lice (both genera *Pediculus* and *Pthirus*) seem to represent the oldest permanent ectoparasites of *Homo sapiens* [26,27]. Despite the progress in medical science and development of the civilization, pediculosis capitis is still an important health issue worldwide [1,28,29]. A large variation in the global spread of head lice is observed. As demonstrated by the available data, its prevalence reaches even 64.1% depending on the examined population [9]. Confinement conditions—e.g., orphanages, adult day care centers, military facilities, and prisons—contribute to the persistence of this parasitosis [24,30,31]. In many countries, including Poland, the frequency of *P. humanus capitis* infestation is underestimated, and there are no tools for monitoring the dynamics of its prevalence. When the regulations in Poland required reporting cases of pediculosis capitis, the incidence (cases per 100,000 inhabitants) in 2004, 2006, and 2008 was 529, 897, and 2653, respectively (Figure S1). Despite the clearly growing tendency, the obligatory reporting of pediculosis capitis cases was discontinued in 2008, which currently impedes the assessment of the occurrence of this parasitosis in our country. Only few research articles on head lice infestation in schoolchildren have been published in Poland since then, e.g., pediculosis capitis was reported in 2% of analyzed schoolchildren [19]. The infestation in children from orphanages was at the level of 17.52% in 2009 [23] and from 6.9 to 29.4% (mean 16.3%) in 2013–2016 [25]. The duty of head lice control is now delegated to school principals, who are responsible for combating lice outbreaks in schools. There are no imposed and universal decisions, and inconsistent actions may result in differences in the occurrence of pediculosis capitis in educational institutions. 

Our present and previous studies revealed a frequent occurrence of pediculosis capitis, as cases of infestation were noted in 87.5% of the surveyed schools. Similar to other studies of this type, the present observations indicate a higher prevalence of head lice in girls, although the relationship between *P. humanus* infestation and the sex of the children turned out to be statistically insignificant [29,32,33]. In the present study, the highest prevalence of infestation (68.0%) was found in the group of the early-school aged children (6–9 years). Other authors indicate similar age groups: 5–7 year-olds [19,34], 4–12 year-olds [11], or 8–11 year-olds [33]. The ease of host-to-host transmission of lice and the role of familial foci in the sustenance of infection sources are evidenced by cases of transmission between siblings recorded in 68.5% of the surveyed schools. Having siblings as one of the factors increasing the probability of *P. humanus capitis* infestation has been indicated by other authors as well [35,36,37]. Dissemination of head louse is facilitated by staying in large groups of people. Our analysis has shown that pediculosis capitis is more frequent in schools with greater numbers of pupils, regardless of their location (rural, suburban, urban). 

Although the present access to information is much easier, there is still a large group of children who do not have any knowledge of this common ectoparasite. In the group surveyed in the present study, 16.7% of the 4970 children had no knowledge of lice, and 5.8–43.3% indicated the sources and routes of *P. humanus capitis* infestation incorrectly. Similar observations were recorded e.g. by researchers from Nigeria, Honduras, and Italy, where 60.2%, approx. 50%, and 57% of respondents answered questions about the mode of transmission of lice incorrectly [15,38,39]. As shown in the present survey, most schools organized educational and information activities on this topic (95.2%), but they were mainly a consequence of an already existing problem (ad hoc action) rather than preventive campaigns. To educate schoolchildren more effectively, it may be necessary to use various forms of knowledge transfer. Various investigations show that both face-to-face contact and interactive media yield similar results in conveying knowledge to children [40]. This is important in the case of children from poorer countries where the prevalence of head lice is high but the access to digital media is limited. An attractive or diversified form of communication may reach a larger group of recipients, although traditional educational campaigns may equally effectively reduce the prevalence of pediculosis capitis in children from poorer regions of the world [41]. As reported by Feinstein et al., an extensive analysis of literature data indicates that, among the multiple factors affecting the population health, education is equally important as the income criterion [42]. Pro-health education can have an impact on health through empowering of behaviors with key importance in the reduction of the prevalence of head lice infestation, e.g., head checking, reporting lice infestations in the immediate environment, and introducing therapeutic activities in accordance with the recommended scheme. In Norway, where seasonal educational campaigns on lice are conducted, members of over 70% of the studied households informed their school, family, and friends about their head lice infestation, and as many as 99.9% declared that they treated pediculosis capitis in accordance with authority recommendations [28]. Analyses conducted in other countries—e.g., Honduras or Nigeria—revealed that treatment was administered in 50% and 44.4–78.2% of pediculosis capitis cases, respectively (depending on the infestation status) [15,38].

The role of parents and teachers is essential in the complete picture of the state of knowledge about pediculosis. Analyses conducted in many countries show no parents’ basic epidemiological knowledge of pediculosis capitis, as in the group of children. Additionally, their awareness of prevention and treatment needs to be improved [39,43,44]. Adults that intend to learn about pediculosis on their own often use unreliable sources of knowledge; therefore, information campaigns based on reliable sources are still necessary [39,44]. Control strategies implemented in schools, which should include education of teachers and parents, have been presented by Speare [45]. As noted by Tappeh, all parents should play a more effective role, and simple and effective training on pediculosis should be useful for periodic examination of children and prophylaxis [32]. Educational campaigns for parents can highlight the importance of periodic head inspections and persuade them to give permission for checkups of their children. Of the schools surveyed in the present study, 58.5% received all parents’ consent for inspection of their child’s head, which implies that still a lot must be changed in this field. As suggested by Saraswat et al. [34], parental education may contribute to reduction of the chance of contracting pediculosis by the child, as it increases compliance with hygienic care standards and the chances of medical intervention in these families. Educating adults is especially important in countries where the burden of the therapeutic process lies mainly with parents and is not supported by health care professionals. In such a case, the lack of knowledge of the recommended therapeutic regimen may result in treatment failure or may contribute to the development of insect resistance in case of overuse of pediculocides [46]. A perspective in lice control seems to lie in noninsecticidal agents, e.g., dimethicone (suffocant) and isopropyl myristate acting through exoskeleton dissolution. They are highly effective substances, and the development of lice resistance is unlikely; however, their use is limited by the patient’s age and relatively high price [47,48,49]. Due to the difficult diagnosis (inspection methods are less effective than the use of special louse combs) and the ease of transmission of lice by asymptomatic carriers, other family members and co-inhabitants should be thoroughly examined and monitored. According to the current recommendations, only infested individuals should be treated [50,51]. An important issue in the treatment of head lice is the choice of an appropriate agent against both the active stages (nymphs and imagines) and eggs, with the lowest possible toxic properties and adapted to the patient’s age and coexisting diseases—e.g., bronchial asthma, allergies, or skin diseases. Furthermore, it is important to follow the manufacturer’s recommendations on the method of application of the preparation and the number of repetitions of the treatment to achieve final elimination of the infestation. In the case of single-use preparations, the head should be inspected on the 1st and 10th day after treatment. In the case of formulations that have to be applied twice, it is recommended that patients have a head inspection one day after the last treatment (usually on the 7th or 10th day). The presence of nits does not mean that the therapy is ineffective, as dead eggs and eggshells may remain attached to the hair for at least 8 months [51].

Pediculosis capitis may be an emotional rather than clinical problem for many individuals. *P. humanus capitis* infection in both children and adults may cause negative feelings and emotions such as disgust, horror, sadness, and fear of being humiliated [44,52,53]. In our study, a large proportion of the surveyed children (26.6%) believe that pediculosis capitis affects dirty and neglected children. Therefore, it is not surprising that only 5% of the surveyed children would inform their friends about their pediculosis problem. It is still an embarrassing topic and little has changed over the years [54].

A positively surprising response is the children’s willingness to broaden their knowledge of pediculosis (57.1%). The statistical analysis showed that mainly the 6–12 year-old children declared the readiness to learn (*p* < 0.0001). This is a valuable indication of the target group for training in this field. Shaping healthy behaviors is easier and more effective in childhood and adolescence, as pupils do not yet have established incorrect attitudes and practices. The effectiveness of such educational activities has been reported by other authors [41,55]. The greater percentage of girls who want to expand their knowledge of head lice may be related to the fact that they are more often affected by this parasitosis. Noteworthy, in the group of younger children from primary schools, schoolgirls are more focused on their health than their male peers [56]. The lesser interest in expanding knowledge declared by the 13–17-year-olds observed in the present study was not associated with a higher level of knowledge of the sources and routes of *P. humanus capitis* transmission. Health education prepares children to take more responsibility for their health, and the lack of interest in this field declared by the adolescents is a disturbing phenomenon also observed by other authors [57]. 

In conclusion, the lack of legal obligations to report pediculosis outbreaks in Poland has a negative impact on the rate and scope of therapeutic activities. This issue is especially important in the case of schoolchildren, as the transmission of this parasitosis is supported by long-term contact in large groups. Educational campaigns targeted at families with early school- and school-aged children should be conducted regularly by appropriately prepared teams rather than in response to an increase in the prevalence of head lice. Education, screening programs, and due action in response to the existing needs may become the key steps towards effective reduction of the prevalence of *P. humanus capitis* in the school environment. This requires systemic changes based on relevant legal regulations.

The first and necessary step to improve the situation is to change the public perception of pediculosis capitis from a hygienic problem to a health issue.

## Figures and Tables

**Table 1:** Descriptive statistics of the number of schoolchildren in the analyzed schools and the result of the Kruskal–Wallis test.

Location of School	*n*	Number of Schoolchildren					H	*p*
		M	SD	Mdn	Min.	Max.		
Rural school	50	133.2	100.8	97.5	16	452	73.33	<0.0001
Suburban school	13	270.5	191.4	212.0	120	830		
Urban school	105	491.8	268.4	476.0	44	1300		

M—mean; SD—standard deviation; Mdn—median; H—value of Kruskal–Wallis test; *p*—probability level.

**Table 2:** Location of the school, prevalence of head lice infestation, and results of the ML (maximum likelihood) chi-square test.

Were Any Cases of Head Lice Reported in The School in 2014–2018?	Location						χ^2^	df	*p*
	Rural School *n* = 50		Suburban School *n* = 13		Urban School *n* = 105				
	*n*	%	*n*	%	*n*	%			
No	12	24.0	1	7.7	8	7.6	7.87	2	0.0196
Yes	38	76.0	12	92.3	97	92.4			

χ^2^—value of the chi-square test; df—degrees of freedom; *p*—probability level; *n* = number of schools.

**Table 3:** Degree of difficulty in pediculosis capitis control versus the frequency of information campaigns and the results of the Fp test comparing frequencies k (multiple responses).

How often Are the Educational Activities Carried Out?	The Solution to the Pediculosis Capitis Issue in Your School Was…						*p*
	Easy/Quite Easy *n* = 70		Difficult/Quite Difficult *n* = 62		Difficult To Say *n* = 23		
	*n*	%	*n*	%	*n*	%	
1 × per school year	26	37.1	21	33.9	5	21.7	0.3768
1 × per semester	12	17.1	23	37.1	8	34.8	0.0245
When the problem occurs	48	68.6	40	64.5	16	69.6	0.8528

χ^2^—value of the chi-square test; df—degrees of freedom; *p*—probability level; *n*—schools that declared to organize educational campaigns.

**Table 4:** Institutions supporting the surveyed schools in limitation of the pediculosis capitis occurrence.

Is The School Supported by Other Institutions in Pediculosis Control? ^1^	*n*	% [*N* = 168] *
Provincial Department of Education	14	8.3
Sanitary-Epidemiological Station	65	38.7
Ministry	0	0.0
Other	15	8.9
No	61	36.3
Total responses	155	-

^1^ multiple-choice question; * percentage calculated for all schools; *n*—number of responses; *N* = number of surveyed schools.

## Data Availability

The data presented in this study are available on request from the corresponding authors.

## Supplementary Materials

The following supporting information can be downloaded at: https://www.mdpi.com/article/10.3390/jcm11030783/s1, Figure S1: Pediculosis in Poland, in the last five years when the regulations required to report its cases to the State Sanitary Inspection (source: Infectious diseases and poisonings in Poland 2004–2008, National Institute of Public Health, National Institute of Hygiene, Department of Epidemiology, Chief Sanitary Inspectorate, Department of Communicable Diseases Control).
