# Supplementary material for: Head Lice Infestation in Schoolchildren, in Poland—Is There a Chance for Change?
Source: J Clin Med. 2022 Jan 31;11(3):783. doi: 10.3390/jcm11030783 (PMC8837132; doi:10.3390/jcm11030783)
Supplement: Supplementary file 1 [file jcm-11-00783-s001.zip › jcm-1532624-supplementary.pdf]

Supplementary files

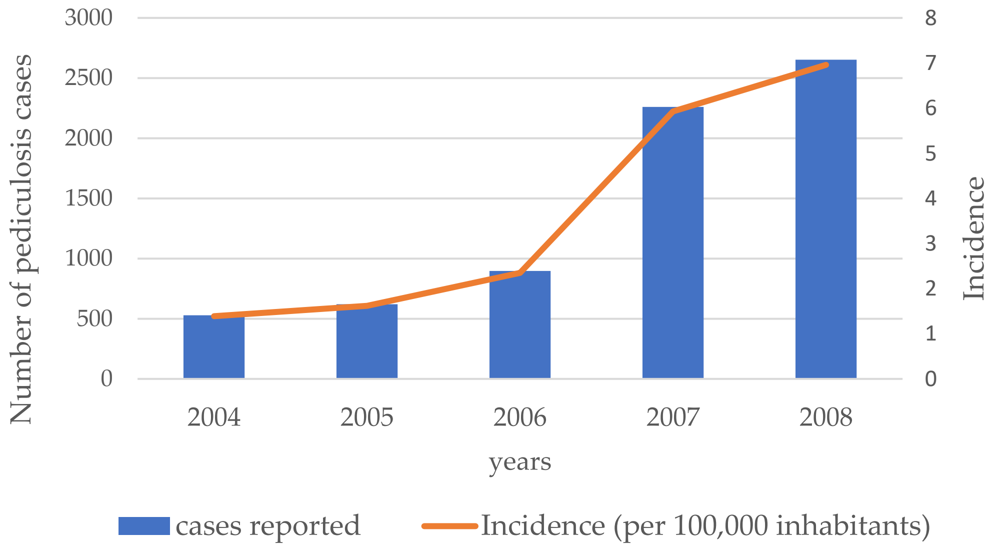

Figure S1. Pediculosis* in Poland, in the last five years when the regulations required to report its cases to the State Sanitary Inspection (source: Infectious diseases and poisonings in Poland 2004-2008, National Institute of Public Health, National Institute of Hygiene, Department of Epidemiology, Chief Sanitary Inspectorate, Department of Communicable Diseases Control). * The reports do not differentiate between pediculosis capitis, pediculosis corporis, and phthiriasis.
